# First report of a Chinese strain of coxsackie B3 virus infection in a newborn in Germany in 2011: a case report

**DOI:** 10.1186/1752-1947-8-164

**Published:** 2014-05-27

**Authors:** Sebastian Ronellenfitsch, Julia Tabatabai, Sindy Böttcher, Sabine Diedrich, David Frommhold, Susanne Schubert-Bast, Johannes Poeschl, Paul Schnitzler

**Affiliations:** 1 Division of Neonatology, Department of Pediatrics, University of Heidelberg, Heidelberg, Germany; 2 Department of Infectious Diseases, Virology, University of Heidelberg, Heidelberg, Germany; 3 National Reference Centre for Poliomyelitis and Enteroviruses, Robert Koch Institute, Berlin, Germany; 4 Division of Pediatric Neurology, Department of Pediatrics, University of Heidelberg, Heidelberg, Germany

**Keywords:** Coxsackievirus B3, Enterovirus, PCR, Viremia

## Abstract

**Introduction:**

Enteroviruses commonly encounter babies and children and infections present in a wide variety of symptoms ranging from asymptomatic infection, benign illness, and aseptic meningitis, hand-foot-and-mouth disease to severe life-threatening disease. Some newborns develop severe disease in the first 2 weeks of life and long-term sequelae may occur among survivors.

**Case presentation:**

We present a case report of a Caucasian newborn baby boy with severe encephalitis and systemic coxsackievirus B3 infection. The coincidence of maternal infection as well as previous mild respiratory illness in his sister suggests either prenatal or horizontal postnatal transmission. An electroencephalogram showed a severe pathologic pattern with theta-delta-rhythm and spike-wave complexes on both hemispheres. We also observed an unusual prolonged viremia for a period of 6 weeks. Due to the lack of specific antiviral treatment options, the supportive management included ventilation and medical treatment of seizures. Phylogenetic analysis revealed a genogroup D2 virus previously exclusively detected in China and now described in Europe for the first time.

**Conclusions:**

Enteroviral infection is an important differential diagnosis in neonatal encephalitis. Prolonged viremia must be taken into account and might correlate with disease severity. The newly observed enterovirus genotype D2 is spreading from Asia to other continents.

## Introduction

Enteroviruses commonly infect children, with syndromes ranging from asymptomatic infection and benign illness, aseptic meningitis, to severe life-threatening disease [1]. Some newborns develop severe disease in the first 2 weeks of life and long-term sequelae may occur among survivors. Risk factors and clinical features associated with severe disease involve early onset of illness within the first few days of life [2]. Enteroviruses include more than 260 different types of pathogens including polioviruses, echoviruses, coxsackieviruses and rhinoviruses. Illness encompasses a wide spectrum of symptoms from mild fever to upper respiratory tract infections, rash, aseptic meningitis, severe myocarditis, encephalitis, and paralytic disorders. Enteroviruses are transmitted primarily via a fecal-oral route and respiratory aerosols [3]. Coxsackie A viruses have been associated with mild clinical symptoms such as flu-like illness and meningitis and hand-foot-and-mouth disease. By contrast, coxsackie B viruses may cause pancreatitis, hepatitis, aseptic meningitis, myocarditis/pericarditis, and type 1 diabetes as well as neonatal sepsis [4-6]. In 2006, six neonates with enteroviral meningoencephalitis were described [7]. Five babies presented with prolonged seizures, and one presented with systemic enteroviral disease. Cranial ultrasonography showed increased echogenicity in the periventricular white matter, and magnetic resonance imaging (MRI) confirmed mild to severe white matter damage in all babies. Recent case reports describe a full-term neonate with coxsackie B2 infection presenting with meningoencephalitis with seizures, and lesions in the white matter, and a fatal case of newborn coxsackie B1 virus infection with apnoeic episodes and desaturations [8,9]. In these cases, an electroencephalogram (EEG) revealed multifocal epileptiform discharges, and a cranial MRI showed multiple lesions, respectively.

## Case presentation

We report here a full-term Caucasian newborn boy, who was delivered by Caesarean section at 38 weeks of gestation in 2011. Within the last weeks of a previously unremarkable pregnancy, his mother showed signs of infection. His 2-year-old sister presented with a common cold within the week of his birth. On the first day postpartum he showed a macular exanthema on his trunk and face. On day 5 postpartum, he showed a decline in his general condition and phases of severe apnea. Hence, he was admitted to a neonatal intensive care unit. Positive airway pressure ventilation was performed for a period of 24 hours followed by spontaneous breathing of the neonate. Broad spectrum antibiotic therapy including gentamicin and ampicillin was started.

**Figure 1:**
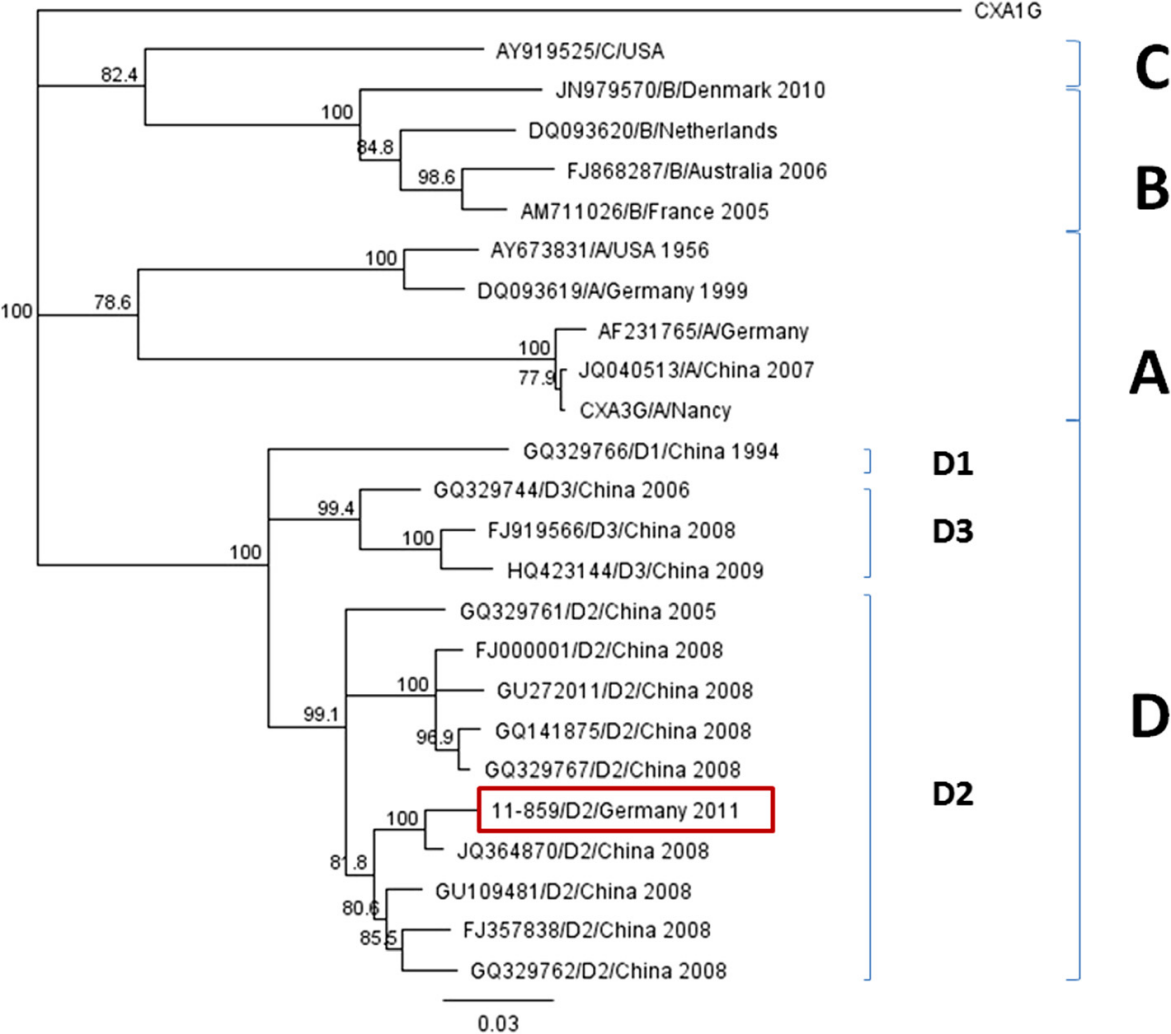
**Phylogenetic analysis of coxsackievirus B3 strains based on 804 nucleotides *VP1 *sequence (according to nucleotide position 2469 – 3272 of coxsackievirus B3 prototype Nancy CXA3G) using representative strains in the GenBank database.** Geneious 6.1.1 software was used to construct the neighbor-joining tree with CoxB1 as outgroup strain (CXA1G). The strain identified in this case report is indicated by a rectangle.

In the course of treatment, he showed signs of partial seizures and an EEG performed on day 7 postpartum indicated a severe pathologic pattern with theta-delta-rhythm and spike-wave complexes on both hemispheres. An intermitted burst-suppression pattern was detected in slow sleep and levetiracetam therapy was initiated. Follow-up EEGs continued to show multifocal spike-wave complexes and burst-suppression. A neurological examination showed a severe muscle hypotonia but no signs of focal neurological deficits. A cardiorespirogram revealed central apnea with desaturations and bradycardias. Due to the clinical picture of encephalitis, a lumbar puncture was performed, showing an elevation of his cell count (29 leucocytes/μL) and protein level (1140g/L). Cerebrospinal fluid (CSF) bacterial cultures were sterile and antibiotic treatment was stopped accordingly. Epstein–Barr virus and herpes simplex virus were not detected in his CSF, but enterovirus ribonucleic acid (RNA) was positive by polymerase chain reaction (PCR). The genetic relationship of the virus was revealed by sequencing the *VP1* gene. This analysis showed that this virus isolate is closely related to coxsackievirus B3 (CV-B3) detected in China in 2008 and belonged to D2 genogroup (Figure 1). The accession number of our isolate 11-859-10 is KJ400861. Retrospectively, all available blood and urine samples were analyzed for enterovirus RNA. In two urine samples and in several serum samples up to week 6 postpartum, enterovirus RNA was still detectable indicating a generalized enterovirus infection with prolonged viremia.

The patient’s seizures ceased rapidly after initiation of levetiracetam medication. A cranial MRI on day 5 revealed a deficiency of white matter with subsequent ventriculomegaly which decreased but was still observed in a follow-up MRI at the age of 3 months. During the further course of treatment anti-convulsive therapy could be stopped since the following EEGs revealed a gradual improvement with cessation of epilepsy typical potentials. Nevertheless, he still showed muscle hypotonia at the age of 1 year.

## Discussion

Perinatal acquired enterovirus infections may lead to severe disease such as meningitis or encephalitis. Recently, two cases of neonatal enterovirus meningitis were reported and etiologically the parents probably transmitted the infection during rooming-in [10]. Severe disease has been described with infection in the first 2 weeks of life and preceding maternal illness peripartum [11]. In the case presented here, maternal infection as well as previous mild respiratory illness in the neonate’s sister preceding the baby’s disease indicates either prenatal or horizontal postnatal transmission.

Central nervous system disease in babies can cause significant long-term morbidity including cerebral palsy and seizures [2,12]. Hypoxia or circulatory compromise as cause for the baby’s neurological state could be excluded as no prolonged hypoxic episodes occurred and myocarditis was not present. Immunoglobulin has been used as a therapeutic agent for neonates; however, clinical efficacy has not been proven. Specific antiviral therapy is not available. Therefore, treatment is supportive and tailored to organ dysfunction including ventilation and medical treatment of seizures [13].

Whereas the diagnosis of enterovirus infection is traditionally performed by viral isolation in cell culture, PCR has been demonstrated to be highly sensitive, specific and rapid [14-16]. We observed an unusual prolonged viremia for a period of 6 weeks, whereas viremia usually lasts only for a few days [17]. Duration and level of detectable viral RNA in blood specimens are positively correlated with disease severity [11]. Most variable regions of the enterovirus genome are within the gene coding for the capsid protein VP1. In 2008, an outbreak of aseptic meningitis due to coxsackie B3 virus genogroup D2 was described for Shandong Province in China [18]. Phylogenetic analysis revealed that the CV-B3 identified in the presented case is closely related to the Chinese outbreak strain and has so far not been reported in Europe.

## Conclusions

Enterovirus infections are of clinical relevance during the neonatal period. In particular, in the typical season, enteroviral infections may be an important differential diagnosis in neonatal encephalitis. We report a newborn presenting with encephalitis and systemic coxsackie B3 virus infection. In addition to the CSF, viral RNA was also detectable in urine and serum samples. The isolated virus is closely related genetically to an enterovirus type previously unique to Eastern China and is now spreading from Asia to Europe.

## Consent

Written informed consent was obtained from the patient’s parents. A copy of the written consent is available for review by the Editor-in-Chief of this journal.

## Abbreviations

CSF: Cerebrospinal fluid; CV-B3: Coxsackievirus B3; EEG: Electroencephalogram; MRI: Magnetic resonance imaging; PCR: Polymerase chain reaction; RNA: Ribonucleic acid.

## Competing interests

The authors declare that they have no competing interests.

## Authors’ contributions

SR, DF, SSB and JP analyzed and interpreted the patient data regarding enterovirus infection. SB and SD performed sequence analysis and sequence comparison. SR, JT and PS wrote the manuscript. All authors read and approved the final manuscript.

## References

[B1] Zaoutis T Klein JD Enterovirus infections Pediatr Rev 1998 19 183 191 10.1542/pir.19-6-183 9613170

[B2] Abzug MJ Presentation, diagnosis, and management of enterovirus infections in neonates Paediatr Drugs 2004 6 1 10 10.2165/00148581-200406010-00001 14969566

[B3] Pallansch MR Oberste MS Whitton JL Polioviruses, coxsackieviruses, echoviruses, and newer enteroviruses Fields Virology 2013 6 490 530

[B4] Rotbart HA Brennan PJ Fife KH Romero JR Griffin JA McKinlay MA Hayden FG Enterovirus meningitis in adults Clin Infect Dis 1998 27 896 898 10.1086/517162 9798053

[B5] Oikarinen S Martiskainen M Tauriainen S Huhtala H Ilonen J Veijola R Simell O Knip M Hyöty H Enterovirus RNA in blood is linked to the development of type 1 diabetes Diabetes 2011 60 276 279 10.2337/db10-0186 20943747 PMC3012181

[B6] Fuschino ME Lamson DM Rush K Carbone LS Taff ML Hua Z Landi K St. George K Detection of coxsackievirus A10 in multiple tissues of a fatal infant sepsis case J Clin Virol 2012 53 259 261 10.1016/j.jcv.2011.12.011 22209288

[B7] Verboon-Maciolek MA Groenendaal F Cowan F Govaert P van Loon AM de VriesL S White matter damage in neonatal enterovirus meningoencephalitis Neurology 2006 66 1267 1269 10.1212/01.wnl.0000208429.69676.23 16636251

[B8] Hirata O Ishikawa N Mizoguchi Y Nakamura K Kobayashi M A case of neonatal coxsackie B2 meningo-encephalitis in which serial magnetic resonance imaging findings reveal the development of lesions Neuropediatrics 2011 42 156 158 21877311 10.1055/s-0031-1285876

[B9] Brecht M Jyoti R McGuire W Chauhan M A case of neonatal coxsackie B virus brainstem encephalitis J Paediatr Child Health 2010 46 699 701 10.1111/j.1440-1754.2010.01907.x 21077981

[B10] Groneck P Jahn P Schuler-Lüttmann S Beyrer K Neonatal enterovirus meningitis: transmission via parents during rooming-in and current epidemiology in Germany Z Geburtsh Neonatol 2011 215 1 5 10.1055/s-0030-1255024 21351051

[B11] Yen M-H Tsao K-C Huang Y-C Huang C-G Huang Y-L Lin R Chang M-L Huang C-C Yan D-C Lin T-Y Viral load in blood is correlated with disease severity of neonatal coxsackievirus B3 infection: early diagnosis and predicting disease severity is possible in severe neonatal enterovirus infection Clin Infect Dis 2007 44 e78 e81 10.1086/515399 17443457

[B12] Kaplan MH Klein SW McPhee J Harper RG Group B coxsackie virus infections in infants younger than three months of age: a serious childhood illness Rev Infect Dis 1983 5 1019 1032 10.1093/clinids/5.6.1019 6318288

[B13] Bryant PA Tingay D Dargaville PA Starr M Curtis N Neonatal coxsackie B virus infection – a treatable disease? Eur J Pediatr 2004 163 223 228 10.1007/s00431-004-1408-y 14986123

[B14] Diedrich S Schreier E Aseptic meningitis in Germany associated with echovirus type 13 BMC Infect Dis 2001 1 14 10.1186/1471-2334-1-14 11591222 PMC57743

[B15] McWilliam Leitch EC Harvala H Robertson I Ubillos I Templeton K Simmonds P Direct identification of human enterovirus serotypes in cerebrospinal fluid by amplification and sequencing of the VP1 region J Clin Virol 2009 44 119 124 10.1016/j.jcv.2008.11.015 19135410

[B16] Reinheimer C Rabenau H Berger A Doerr HW Diagnostic of neurotropic enteroviruses in children with CSF and/or stool: virus isolation by cell culture or PCR? Klin Padiatr 2011 223 221 226 10.1055/s-0031-1273725 21574127

[B17] Mueller S Wimmer E Cello J Poliovirus and poliomyelitis: A tale of guts, brains, and an accidental event Virus Res 2005 111 175 193 10.1016/j.virusres.2005.04.008 15885840

[B18] Tao Z Song Y Li Y Liu Y Jiang P Lin X Liu G Song L Wang H Xu A Coxsackievirus B3, Shandong province, China, 1990–2010 Emerg Infect Dis 2012 18 1865 1867 10.3201/eid1811.120090 23092737 PMC3559141

